# Assessing Whether Meditation Improves Quality of Life for Adolescent Girls With Polycystic Ovary Syndrome: Protocol for a Randomized Controlled Trial

**DOI:** 10.2196/14542

**Published:** 2020-01-28

**Authors:** Erandi Hewawasam, Leah Brennan, Lynne Giles, Mary Louise Hull, Asha Short, Robert Norman, Alexia S Peña

**Affiliations:** 1 Discipline of Paediatrics Robinson Research Institute University of Adelaide Adelaide Australia; 2 Department of Endocrinology and Diabetes Women’s and Children’s Hospital North Adelaide Australia; 3 School of Behavioural and Health Sciences Australian Catholic University Fitzroy Australia; 4 Centre for Eating, Weight and Body Image Melbourne Australia; 5 School of Psychology and Public Health La Trobe University Wodonga Australia; 6 School of Public Health Faculty of Health and Medical Sciences University of Adelaide Adelaide Australia; 7 Department of Obstetrics and Gynaecology Women’s and Children’s Hospital North Adelaide Australia; 8 Obstetrics and Gynaecology Robinson Research Institute The University of Adelaide North Adelaide Australia

**Keywords:** meditation, quality of life, polycystic ovary syndrome, adolescent

## Abstract

**Background:**

Polycystic Ovary Syndrome (PCOS) is a common endocrine condition characterized by irregular periods and hyperandrogenism. Adolescents with PCOS have impaired quality of life (QOL) and increased psychological distress. Transcendental Meditation (TM) is a well-established self-management strategy that has been used to improve stress and well-being. A meta-analysis of TM trials has shown beneficial effects on stress and blood pressure in adults. Recent data are suggesting that another self-management strategy called a mindfulness stress management program has a role in improving QOL in women with PCOS, but there are no studies in adolescents.

**Objective:**

This study aims to evaluate the effect of TM on QOL and psychological distress in adolescent girls with PCOS.

**Methods:**

This study is a randomized controlled trial that will be conducted over eight weeks at the Women’s and Children’s Hospital in Adelaide, South Australia, to determine the effect of TM on QOL and psychological distress in adolescent girls (aged 12-20 years) with PCOS. A total of 40 girls will be randomized into either the TM (n=20) or control group (n=20). The TM group will be asked to practice TM in a comfortable sitting position with the eyes closed, for 15 minutes twice daily over eight weeks. The control group will be asked to sit quietly for 15 minutes twice daily for eight weeks. The primary outcomes are any effects on improving QOL and psychological distress, and the secondary outcomes are any effects on lowering blood pressure and salivary cortisol levels.

**Results:**

The recruitment of study participants began in May 2019 and is expected to be completed by June 2020. It is expected that the adolescent girls with PCOS practicing TM over eight weeks will have a significant improvement in QOL and psychological distress compared to adolescents in the control group. Also, it is expected that adolescent girls in the TM group will have lower salivary cortisol levels and lower blood pressure.

**Conclusions:**

This study will be the first to evaluate the effect of TM on QOL in adolescent girls with PCOS. The study will provide valuable information on a potential self-management strategy to improve QOL and well-being in adolescent girls with PCOS.

**Trial Registration:**

Australian New Zealand Clinical Trials Registry (ANZCTR) ACTRN1261900019010; https://www.anzctr.org.au/Trial/Registration/TrialReview.aspx?id=376657&amp;amp;isReview=true

**International Registered Report Identifier (IRRID):**

PRR1-10.2196/14542

## Introduction

### Polycystic Ovary Syndrome

Polycystic Ovary Syndrome (PCOS) is the most common endocrine condition and affects 8-13% of women of reproductive age [1] and up to 6% of adolescent girls [2], depending on the diagnostic criteria used and the population studied [3-8]. Adolescent PCOS is characterized by irregular menstrual cycles and at least one of the following signs of hyperandrogenism (hirsutism, acne or hyperandrogenemia) [3-8]. PCOS is associated with increased weight, difficulties losing weight, insulin resistance, metabolic syndrome, infertility, and psychological distress (eg, depression, anxiety, and stress) [3,5,7-9].

### Quality of Life in Polycystic Ovary Syndrome

Two systematic reviews have shown that women with PCOS have reduced quality of life (QOL) [9,10]. Women with PCOS are also at increased risk of psychological distress [5,7,8] and have reported higher scores in the Depression, Anxiety, and Stress Scale (DASS) questionnaire compared to women without PCOS [11]. Similarly, adolescent girls with PCOS are more likely to have anxiety and depression than age-matched healthy adolescents [12]. A mixed-methods study (including quantitative and qualitative data) showed that adolescent girls with PCOS are at an increased risk of depression [13]. Also, adolescent girls with PCOS have reduced QOL according to cross-sectional quantitative and qualitative studies [12,14,15].

General health and its perception, behavior, physical functioning, and the family activities domains of QOL are lower in adolescent girls with PCOS in comparison to healthy adolescents [12]. Specific symptoms of PCOS, such as hirsutism, excess weight, irregular menstrual cycles, and infertility problems, can affect QOL, as demonstrated by lower scores in the specific QOL questionnaire for PCOS [16]. Increased weight and body perceptions are essential contributors to reduced QOL as well as fears and concerns about future infertility [17,18]. In a Bulgarian study of women with PCOS, it was shown that hirsutism (predominantly in those <25 years of age), excessive weight gain, and infertility (in those >25 years of age) were independently associated with decreased QOL [19].

### Strategies to Improve Quality of Life in Polycystic Ovary Syndrome

Most management strategies used in adolescent girls with PCOS have targeted symptoms such as irregular menstrual cycles, acne, and hirsutism, or have addressed excess weight and insulin resistance [5,7,8]. There are limited data directly evaluating interventions addressing psychological comorbidities in PCOS, such as reduced QOL and psychological distress [5,7,8].

In women with PCOS, there is increasing evidence for the role of complementary and alternative medicine [20] and self-management strategies, such as mindfulness-based stress reduction, in the management of stress, and improving QOL [21-23]. In adolescent girls with PCOS, however, there have only been two studies conducted that evaluated strategies to improve QOL [24,25]. One study was a small open trial with no control group that used cognitive behavioral therapy to target lifestyle goals, family functioning, and the effects of having PCOS [24]. This study showed a statistically significant improvement in health-related quality of life from a mean of 77 points (SD 20) from baseline to a mean of 82 points (SD 27) post-intervention [24]. In the other study, participants received a lifestyle modification program (ie, weekly discussions on nutrition and lifestyle changes and weekly structured group exercise sessions) that was associated with a significant improvement from 4.5 to 5.6 points on the specific QOL questionnaire for PCOS, which was calculated by averaging all domains of QOL related to PCOS [25].

### Transcendental Meditation as a Strategy to Improve Quality of Life

Transcendental Meditation (TM) is a sitting meditation technique from ancient Vedic tradition that originated in India and has spread worldwide since the 1950s [26]. It is a well-established and easily practiced technique while sitting with the eyes closed in adults for 20 minutes twice daily, and in children for 15 minutes twice daily. The TM technique uses the sound value of a mantra to draw attention within the mind, leading to a relaxed but mentally alert state. Trained instructors taught TM in a standardized manner with careful attention to fidelity of the program by regular review sessions of the TM practice.

TM has been used in adults and adolescents to reduce stress in conditions such as cardiovascular disease and negative school behaviors [27-31]. Meta-analysis of stress reduction trials, including TM, has shown beneficial effects on stress and blood pressure in adults, with systolic and diastolic blood pressures of –4.26 mm Hg (95% CI –6.06 to –2.23) and –2.33 mm Hg (95% CI –3.70 to –0.97) respectively, compared to control groups [30,32], with increasing use recently [33]. Sitting meditation practices, including TM and mindfulness, may have some beneficial effects in physiological, psychological, and behavioral conditions in children and adolescents [34]. Few randomized controlled trials in adolescents have demonstrated beneficial effects of TM on school behavior and blood pressure [27,35]. Also, TM has shown beneficial effects on cardiovascular outcomes, as measured by left ventricular mass index in a group of prehypertensive adolescents in comparison to the control group (–2.6 gm/ht^2.7^ versus 0.3 gm/ht^2.7^) [36].

To date, no trials have evaluated the effect of TM on women or adolescent girls with PCOS. This proposal aims to evaluate TM as a novel strategy, which is easy to implement once learned, to improve QOL and psychological distress in adolescent girls with PCOS using a randomized controlled trial.

### Aims and Hypotheses

The primary objectives are to determine the effect of TM over eight weeks on QOL and psychological distress in adolescent girls with PCOS. The secondary objectives are to determine the effect of TM over eight weeks on salivary cortisol levels and blood pressure in adolescent girls with PCOS. We hypothesize that the adolescent girls with PCOS practicing TM over eight weeks will have a significant improvement in QOL and psychological distress in comparison to adolescents in the control group. We also hypothesize that those in the TM group will have lower salivary cortisol levels and lower blood pressure.

## Methods

### Study Design, Approval, and Registration

**Figure 1:**
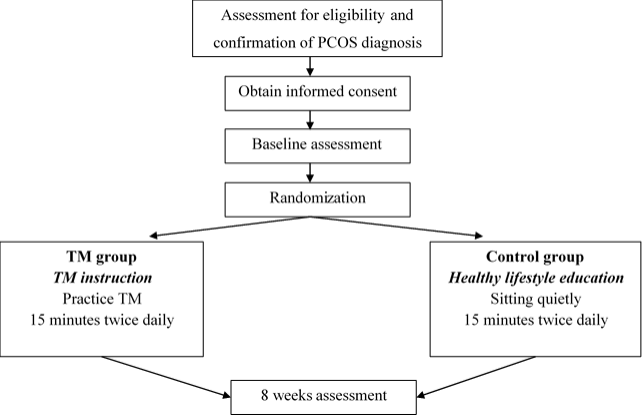
Study design flowchart. PCOS: polycystic ovary syndrome.

This is a randomized controlled trial that will be conducted over eight weeks at the Women’s and Children’s Hospital in Adelaide, South Australia. A flowchart of the study is depicted in Figure 1. The trial has been approved by the Women’s and Children’s Hospital Research Ethics Committee (HREC/18/WCHN/168, protocol version 2.0, dated January 11, 2019), and any modifications to the protocol will be sent to the Ethics Committee. This study has been registered with the Australian New Zealand Clinical Trials Registry (ACTRN12619000190101).

### Recruitment

A total sample of 40 adolescent girls, aged 12-20 years old and with a confirmed diagnosis of PCOS, as per the recent international evidence-based guidelines [5,7,8], will be recruited by authors from various outpatient clinics (adolescent gynecology and endocrine clinics, pediatric endocrine clinics, and gynecology clinics). These include clinics at the Women’s and Children’s Hospital in South Australia, from private gynecology clinics in South Australia, from the Polycystic Ovary Syndrome Association of Australia (POSAA) consumer support groups, Health consumer groups in South Australia, and the Women’s and Children’s Hospital consumer groups. If the adolescent is older than 16 years of age, written informed consent will be obtained from the adolescent. If the adolescent is under 16 years of age, written informed assent will be obtained from the adolescent, and written informed consent will be obtained from their parent/guardian. Participants can also either consent or not consent to the future use of study data in any other research project, provided the project has the approval of the Women’s and Children’s Hospital Research Ethics Committee.

### Inclusion Criteria

Each adolescent girl must meet the following criteria to be involved in this study: aged between 12-20 years old; diagnosed with PCOS according to the recent, international, evidence-based guideline of diagnostic criteria for PCOS, consisting of a clear definition of irregular menstrual cycles according to time post menarche and at least one of the following signs of hyperandrogenism (moderate to severe acne, hirsutism or hyperandrogenemia [high androgen levels]) after exclusion of other conditions that mimic PCOS [5,7,8]; for participants not recruited at the Women’s and Children’s Hospital, a review by the principal investigator (AP) will be done to confirm PCOS diagnosis as per recent guidelines [5,7,8]; on a stable medical regimen for at least the previous four weeks if on metformin or other hormonal therapy (contraceptive pill, medroxyprogesterone, or spironolactone); willingness and ability to participate in the TM instruction session and practice TM during the study; the willingness of one parent to be involved in facilitating and keeping a diary card of the TM or sitting activities in the control group.

### Exclusion Criteria

Adolescent girls are excluded from the study if they meet any of the following criteria: significant coexisting chronic illness that may have a contributory effect on health-related QOL, such as diabetes (Type 1 or 2), gender dysphoria, or severe anxiety or depression treated with medications; psychological conditions, such as schizophrenia or bipolar disorder; high level of depression (DASS score>20), anxiety (DASS score>15), or stress (DASS score>26) as they will require immediate treatment; regularly practicing the TM or any other meditation/mindfulness program; substance use, as it will interfere with the TM practice; or inability to speak English.

### Randomization

A randomization schedule was generated in Stata 15.1 (StataCorp, College Station, Texas, United States) by an independent statistician (SE). Randomly permuted blocks of size 2 or 4 and an allocation ratio of 1:1 (TM: control) was used. Unique identification codes and random group allocation will be generated for each participant. Sequentially-numbered, opaque, sealed envelopes containing each participants’ group assignment will be created by a pharmacist not involved in the study. The allocation sequence will be unavailable to study investigators. Participants will be allocated to either the TM group or control group according to information contained in the sealed, opaque envelope. An envelope will only be opened after the participant has been enrolled and completed baseline clinical assessments. Analysis of the data will be performed by a statistician who was not involved in the initial randomization process (LG).

### Participant Withdrawal

Any participant may withdraw from the study without giving a reason and without any disadvantage or interference with their care at their treating hospital. Reasons for withdrawal will be noted and reported.

### Intervention

#### Transcendental Meditation

Participants allocated to the TM group will be asked to practice TM for 15 minutes, twice daily, for eight weeks. The instructional sessions for the TM technique will be delivered by a certified and experienced female teacher [37] at either TM Adelaide or the Adelaide South TM Centre in South Australia.

TM will be taught according to a standardized course. Before beginning this course, participants will be asked to attend a one-hour introductory presentation reviewing the origin of TM and the possible benefits of regular practice. The participant and one of the parents are required to attend this introductory session. If the participant is younger than 18 years of age, then the participant’s parent will be required to provide a letter of support stating that they approve of their child’s participation in the standardized course.

After the introductory presentation, there will be a one-on-one personal interview for the participant to provide the teacher with some basic information to aid in the personalization of their instruction (Table 1). On the first day of instruction, the participant will be given a personal mantra (sound or word) that will be used in every meditation. This sound will help draw attention within the mind, leading to a relaxed but mentally alert state. After the initial instruction, the participants will be instructed to practice TM for 15 minutes, twice daily, at home.

Following the personal instruction session, there will be check-up meditation sessions on three consecutive days during the first week and an additional day in week 2 to discuss the participant’s experiences during meditation. This meeting will also assist with any practical issues encountered during practice, such as handling outside noise and timing of the meditation.

Practice review sessions will occur from weeks 3-6, where participants will be able to practice TM in a small group (Table 1). The TM teacher will reinforce the fidelity of the program by encouraging multiple practice review sessions after initial instruction and check-ups. Participants/parents will also complete a diary showing the frequency of meditation practice.

**Table 1:** Instruction, check-up, and practice review sessions for the transcendental meditation group.

Appointment	Timing	Activity	Duration
1	Day 1	Personal instruction to learn transcendental meditation	1 hour
2	Day 2	Check-up, including meditation	1 hour
3	Day 3	Check-up, including meditation	1 hour
4	Day 4	Check-up, including meditation	1 hour
5	Week 2	Check-up, including meditation	1 hour
6	Week 3	Practice review, including meditation	30 minutes
7	Week 4	Practice review, including meditation	30 minutes
8	Week 6	Practice review, including meditation	30 minutes

#### Control Group

Participants in the control group will be asked to sit comfortably for 15 minutes twice daily in a quiet room for eight weeks. Participants will be allowed to read a book, write, or listen to some music. The control group will receive a one-hour, healthy lifestyle education session and healthy lifestyle information via email/phone at similar times to the intervention group (Table 2). Participants/parents will also complete a diary, including sitting activities.

**Table 2:** Healthy lifestyle education session and information for the control group.

Appointments	Timing	Activity
1	Day 1	Healthy lifestyle education session (1 hour)
2	Day 2	Email or phone contact with healthy lifestyle information
3	Day 3	Email or phone contact with healthy lifestyle information
4	Day 4	Email or phone contact with healthy lifestyle information
5	Week 2	Email or phone contact with healthy lifestyle information
6	Week 3	Email or phone contact with healthy lifestyle information
7	Week 4	Email or phone contact with healthy lifestyle information
8	Week 6	Email or phone contact with healthy lifestyle information

### Outcome Measures

Table 3 shows the primary and secondary outcomes that will be measured at baseline (preintervention) and eight weeks (postintervention) at the Women’s and Children’s Hospital.

**Table 3:** Primary and secondary outcome measures.

Outcome measure			Baseline	Eight weeks
**Primary outcomes**				
	Pediatric QOL^a^	✓		✓
	QOL related to PCOS^b^	✓		✓
	DASS^c^	✓		✓
**Secondary outcomes**				
	Salivary cortisol levels (3 samples over a day)	✓		✓
	Blood pressure	✓		✓
	Weight	✓		✓
	Height	✓		✓
	BMI^d^	✓		✓
	BMI *z* score	✓		✓
	Waist circumference	✓		✓
	Hip circumference	✓		✓
	Hirsutism score and acne score	✓		✓
	Review of current symptoms and treatment	✓		✓

^a^QOL: quality of life

^b^PCOS: Polycystic ovary syndrome

^c^DASS: Depression, Anxiety and Stress Scale

^d^BMI: body mass index

### Primary Outcomes

#### Quality of Life

General QOL will be assessed using the Peds QLTM 4.0 generic core scales [38]. The generic core scale consists of 23 items, which will be completed independently by participants and one of their parents. It includes four scales that measure physical, emotional, social, and school functioning.

#### Specific Quality of Life Questionnaire for Polycystic Ovary Syndrome

This questionnaire measures health-related QOL in PCOS over the previous two weeks. It consists of 26 items covering five domains (emotions, body hair, weight, infertility problems, and menstrual problems). Each item is graded with a 7-point scale ranging from 1 (maximum impairment) to 7 (no problems experienced) [16,25].

#### Depression, Anxiety and Stress Scale Questionnaire

This is a self-reported instrument that was designed to evaluate the three emotional states of depression, anxiety, and tension or stress [39]. This will also assist in the evaluation of the severity of symptoms to assess the eligibility of the participants.

### Secondary Outcomes

Salivary cortisol levels will be measured as a marker of stress in adolescents [40]. Salivary cortisol will be collected with salivary cortisol sampling kits (Salivette). Specific instructions will be provided for proper collection. Three salivary cortisol samples will be collected as the cortisol levels change throughout the day (immediately after awakening in the morning, 30 minutes later, and before going to bed). Samples will be collected the day after baseline assessment and the day before the eight-weeks assessment. Salivary cortisol concentrations will be measured by IDS-iSYS Salivary Cortisol assay (Abacus ALS Pty Ltd, Queensland, Australia). Salivary samples will be deidentified before analysis. Salivary samples will not be retained for future use and will be put in the human biohazard bins for appropriate destruction immediately after completion of the analysis.

Also, weight will be measured in light clothing using a Tanita BC-418 segmental Body composition analyzer (Tanita, Tokyo, Japan). Height will be measured on a wall-mounted stadiometer (to 0.1 cm). Waist circumference will be measured with a flexible tape (0.5 cm) at the midpoint between the lower margin of the last palpable rib and the top of the iliac crest, and hip circumference will be measured around the widest portion of the buttocks, with the tape parallel to the floor, following World Health Organization guidelines [41]. Body mass index (BMI) percentiles and *z* score will be calculated using the EpiInfo database version 3.2.2 and the Centers for Disease Control and Prevention 2000 standardized reference charts [42]. Blood pressure will be measured using an automatic sphygmomanometer (Omron digital blood pressure monitor, Omron Healthcare, United Kingdom) with an appropriately sized cuff on the left arm, and the mean of 3 consecutive measurements will be recorded. We will also evaluate hirsutism using the modified Ferriman Gallwey score [43], and acne will be assessed using the Global Acne Grading System [44]. Finally, a review of current symptoms and treatment will be documented at baseline and eight weeks posttreatment.

### Adherence

Assessment of adherence to the study will be assessed using the diary that participants and parents complete about their time spent practicing TM or undertaking sitting activities. Also, the TM teacher will reinforce adherence to TM during check-up and practice review sessions.

### Monitoring of Adverse Events

TM is a safe self-management strategy that has been used by many adults around the world, with rarely reported adverse events. These events occurred in individuals with severe psychiatric conditions, but participants with such conditions will be excluded from this study. TM practice can rarely cause mild headaches at the beginning if it is not done correctly. Teachers of the TM technique are trained to correct any difficulties with practice. Any adverse events noted will be reported to the ethics committee and documented.

### Data Management and Statistical Analysis

Data collected during this study will be both hard copy (questionnaires and case report forms) and electronic (excel spreadsheets with participant’s data) files. Hard copy data will be stored securely in the Department of Endocrinology and Diabetes at the Women’s and Children’s Hospital, and electronic data will be stored securely using password protected files. Documents will be retained for 30 years after study completion per current guidelines for data storage.

All data will be analyzed in the deidentified form. Statistical analysis will be performed using the intention-to-treat principle by LG, who will be blinded to the randomization status. A linear mixed-effects model will be used to compare the change in primary outcomes (QOL, specific QOL questionnaire for PCOS, and DASS questionnaires) and secondary outcomes (salivary cortisol levels and blood pressure) between the TM group and control group over the study period. The models will include a random effect for participants to account for the correlation between serial measurements within individual participants. Unadjusted and adjusted analyses will be conducted, with adjustment for important prespecified baseline covariates, including age and BMI (except for the analyses where BMI is the outcome).

While no studies have looked at the effect of TM on QOL in adolescent girls with PCOS, a change in QOL of 21.2 (SD 21.5) will be interpreted as clinically meaningful, as per our previous study in obese adolescents [45]. The sample size was calculated based on a change in QOL of 21.2 (SD 21.5) from baseline to 8 weeks in the TM group, compared to no change in the control group. Therefore, we would require 40 adolescent girls (20 in each treatment group) to have 80% power to detect a difference in the primary outcome of QOL between the TM group and control group (assuming alpha=0.05, and a two-sided statistical test), allowing for 10% attrition based on rates in previous studies that included adolescents in our center [46]. The sensitivity of the effect estimates to missing values will be evaluated using multiple imputation. Analyses will be conducted according to a pre-specified analysis plan using Stata version 15.1 (StataCorp, College Station, Texas, United States).

## Results

Recruitment started in May 2019 and is expected to be completed by June 2020. It is expected that adolescent girls with PCOS practicing TM over eight weeks will have a significant improvement in QOL and psychological distress compared to adolescents in the control group. Also, it is expected that adolescent girls in the TM group will have lower salivary cortisol levels and lower blood pressure. The results from this trial are anticipated to be published by September 2020.

## Discussion

PCOS is the most common endocrine condition affecting women of reproductive age and is associated with many comorbidities, which contribute to impaired QOL and increased psychological distress [3,5,7,8]. Currently, health care professionals have limited strategies to offer to improve the well-being of this population beyond hormonal and metabolic treatments. Evaluating the efficacy of a novel sustainable strategy in the management of PCOS will assist in the overall management of this condition from adolescence and into young adulthood, as once this strategy is learned, it can be practiced for life. Although studies are suggesting that mindfulness may have a role in improving QOL in women with PCOS, there are no studies conducted in adolescents and no studies of TM in women or adolescents with PCOS.

To our knowledge, this is the first study evaluating the effect of TM on QOL and psychological distress in adolescent girls with PCOS. The current study will evaluate a simple, translatable strategy to improve QOL and well-being in adolescent girls with PCOS. TM will be easy to implement in the long term, with no additional costs once the strategy has been learned. This intervention can also be potentially taught in schools as part of classroom activities.

## Abbreviations

BMI: Body mass index
DASS: Depression, Anxiety and Stress Scale
PCOS: Polycystic ovary syndrome
QOL: Quality of life
TM: Transcendental Meditation
